# Association between surgeon age and postoperative complications/mortality: a systematic review and meta-analysis of cohort studies

**DOI:** 10.1038/s41598-022-15275-7

**Published:** 2022-07-04

**Authors:** Yeongin Jung, Kihun Kim, Sang Tae Choi, Jin Mo Kang, Noo Ree Cho, Dai Sik Ko, Yun Hak Kim

**Affiliations:** 1 Department of Medicine, School of Medicine, Pusan National University, Busan, Republic of Korea; 2 Department of Occupational and Environmental Medicine, Kosin University Gospel Hospital, Busan, Republic of Korea; 3 Division of Vascular Surgery, Department of Surgery, Gachon University Gil Medical Center, Incheon, 21565 Republic of Korea; 4 Department of Anesthesiology and Pain Medicine, Gachon University Gil Medical Center, Incheon, 21565 Republic of Korea; 5 Department of Biomedical Informatics, School of Medicine, Pusan National University, Yangsan, 50612 Republic of Korea; 6 Department of Anatomy, School of Medicine, Pusan National University, Busan, Republic of Korea

**Keywords:** Health care, Medical research

## Abstract

The surgical workforce, like the rest of the population, is ageing. This has raised concerns about the association between the age of the surgeon and their surgical outcomes. We performed a systematic review and meta-analysis of cohort studies on postoperative mortality and major morbidity according to the surgeons’ age. The search was performed on February 2021 using the Embase, Medline and CENTRAL databases. Postoperative mortality and major morbidity were evaluated as clinical outcomes. We categorized the surgeons’ age into young-, middle-, and old-aged surgeons. We compared the differences in clinical outcomes for younger and older surgeons compared to middle-aged surgeons. Subgroup analyses were performed for major and minor surgery. Ten retrospective cohort studies on 29 various surgeries with 1,666,108 patients were considered. The mortality in patients undergoing surgery by old-aged surgeons was 1.14 (1.02–1.28, *p* = 0.02) (I^2^ = 80%) compared to those by middle-aged surgeon. No significant differences were observed according to the surgeon’s age in the major morbidity and subgroup analyses. This meta-analysis indicated that surgeries performed by old-aged surgeons had a higher risk of postoperative mortality than those by middle-aged surgeons. Thus, it necessitates the introduction of a multidisciplinary approach to evaluate the performance of senior surgeons.

## Introduction

The global health care system has witnessed remarkable improvement in postoperative morbidities and mortalities in the past 25 years^1^. Nevertheless, there are nearly 4.2 million deaths, within 30 days of surgery worldwide each year, accounting for the third largest reason for death after ischaemic heart disease and stroke^2^. Furthermore, a report suggests the occurrence of adverse events in 14.4% of surgical patients in developed countries, including the USA and Canada^3^.

Improvements in individual surgical performance have been recognised as a cornerstone in delivering safe and quality health care^4^. A recent review of the surgical performance by Maruthappu et al.^5^ showed that an increased volume of cases and years of surgical practice is associated with improved health outcomes, such as recurrent rate, perioperative complications, and mortality. Moreover, a plateau phase or maturation was observed in the surgical learning curve, where the case volume and years of surgical practice no longer resulted in considerable improvements in outcomes. Duclos et al.^6^ reported that surgeons with experience of more than 20 years in conducting thyroidectomy exhibited a significantly increased probability of recurrent laryngeal nerve palsy and hypoparathyroidism and raised a concave association between the outcome and length of experience.

Despite substantial interest in the surgeons’ age and their surgical performance, the association between the surgeons’ age and patients’ outcome has not been widely studied. According to the Association of American Medical Colleges’ physician specialty data report in 2019, the population of active surgeons aged 55 years or older varies across specialities; for example general surgery, 47.5%; orthopaedic surgery, 57.1%; thoracic surgery, 60.1%^7^. Some reports have raised a concern that the surgeons’ performance may decline with ageing as they tend to perform poorly in the recertification examinations and are less likely to have a current knowledge base^8,9^. In this context, we conducted a meta-analysis of studies evaluating the influence of the surgeons’ age on the clinical outcomes, namely, (1) postoperative mortality and (2) major morbidity.

## Methods

### Protocol and registration

This systematic review was conducted according to the PRISMA guideline^10^ (Supplementary Information). The protocol mentioned in this article was registered at PROSPERO (Registration number: CRD42021234343).

### Eligibility criteria

We defined PICOS as “Are there differences in adverse outcomes and mortality according to the age of surgeons in patients undergoing various surgeries?”. We categorised surgeons into young, middle-aged, and old-aged groups and evaluated the influence of the surgeons’ age on the clinical outcomes, like postoperative mortality and major morbidity. Major morbidity was defined as the presence of one of the following events: postoperative complications, revision surgery, or readmission. Only the cohort studies were eligible for inclusion in this study. Papers that combined mortality and major morbidity were excluded. For performing meta-analysis, we excluded papers that express surgeon age as a continuous variable rather than a categorical variable. Papers in which surgeon age was presented as a continuous variable rather than a categorical variable were excluded for performing meta-analysis.

We categorised the surgeons’ age into three groups: young-, middle-, and old-aged surgeons. Although we tried to clearly define the group age range, the criteria for age classification were heterogeneous between studies. The definition of surgeon age in the paper was extracted when the age of surgeon was presented in three categories. The age groups of surgeons were determined by discussion among the authors in papers that included more than three categories.

### Search strategy

The search was performed on 2 February 2021 using the Embase, Medline and CENTRAL databases. The search terms were as follows: (doctor age OR physician age OR surgeon age OR old doctor OR old physician OR old surgeon OR older doctor OR older physician OR older surgeon OR elderly doctor OR elderly physician OR elderly surgeon OR young doctor OR young physician OR young surgeon OR junior doctor OR junior physician OR junior surgeon OR senior doctor OR senior physician OR senior surgeon) AND (risk OR ratio OR hazard OR odds OR prevalence OR incidence OR outcome OR prognosis OR mortality OR morbidity OR death OR survival OR dead OR relapse OR recur OR recurrence OR complication). The search was limited to titles and abstracts. We did not restrict by language or publication year. We included article or article in press type papers. In addition, a manual search was performed to extract grey literatures.

### Selection criteria

The literature search was conducted independently by two authors (KS and JY) and the title and abstract for each study were checked thoroughly. The full-text articles were reviewed by the same authors for inclusion. Any disagreements were resolved through discussion.

### Data extraction

The following data was extracted in the screening phase: title, abstract, journal, author name, and publication year and type. Additional information on the study design, physician age, type of surgery, effect measures, study period, WHO region, number of samples, and data source was extracted through a full-text assessment.

### Summary measures

The number of events and total subjects presented in the paper were used to calculate the odds ratio (OR). If the above values could not be extracted, the unadjusted OR was extracted preferentially over the adjusted OR in the paper. The Hazard ratio was considered equal to the OR for meta-analytic purpose.

### Risk of bias in the individual studies

The Newcastle–Ottawa scale was used to qualitatively assess the risk of bias for the included cohort studies^11^. The authors (YJ and KK) independently assessed the risk of bias of the included studies and verified the quality of the evidence. If there was a discrepancy in the assessment, it was resolved through discussion. The study scores were converted into three categories of ‘good’, ‘fair’, and ‘poor’ according to the Agency for Healthcare Research and Quality standard.

### Statistical analyses

The classification of I^2^ statistics as presented by Higgins et al. was used to evaluate the heterogeneity of the effect measures^12^. The heterogeneity was considered low, moderate, and high for I^2^ values of 25%, 50%, and 75%, respectively. If the heterogeneity exceeded 50%, the random effect method was used; otherwise, the fixed-effect method was used. If an integrated value was required within the study, the calculation was performed using the Higgins method^12^. Forest plots were drawn to clearly visualize synthesized risk. The Review Manager 5.4 software was used to synthesize results.

For major morbidity, the subgroup analysis was conducted separately as major and minor surgery. Based on the principles proposed by Small (1965), we categorized each type of surgery into major and minor surgery through discussion among the authors^13^.

## Results

### Study selection and characteristics

**Figure 1:**
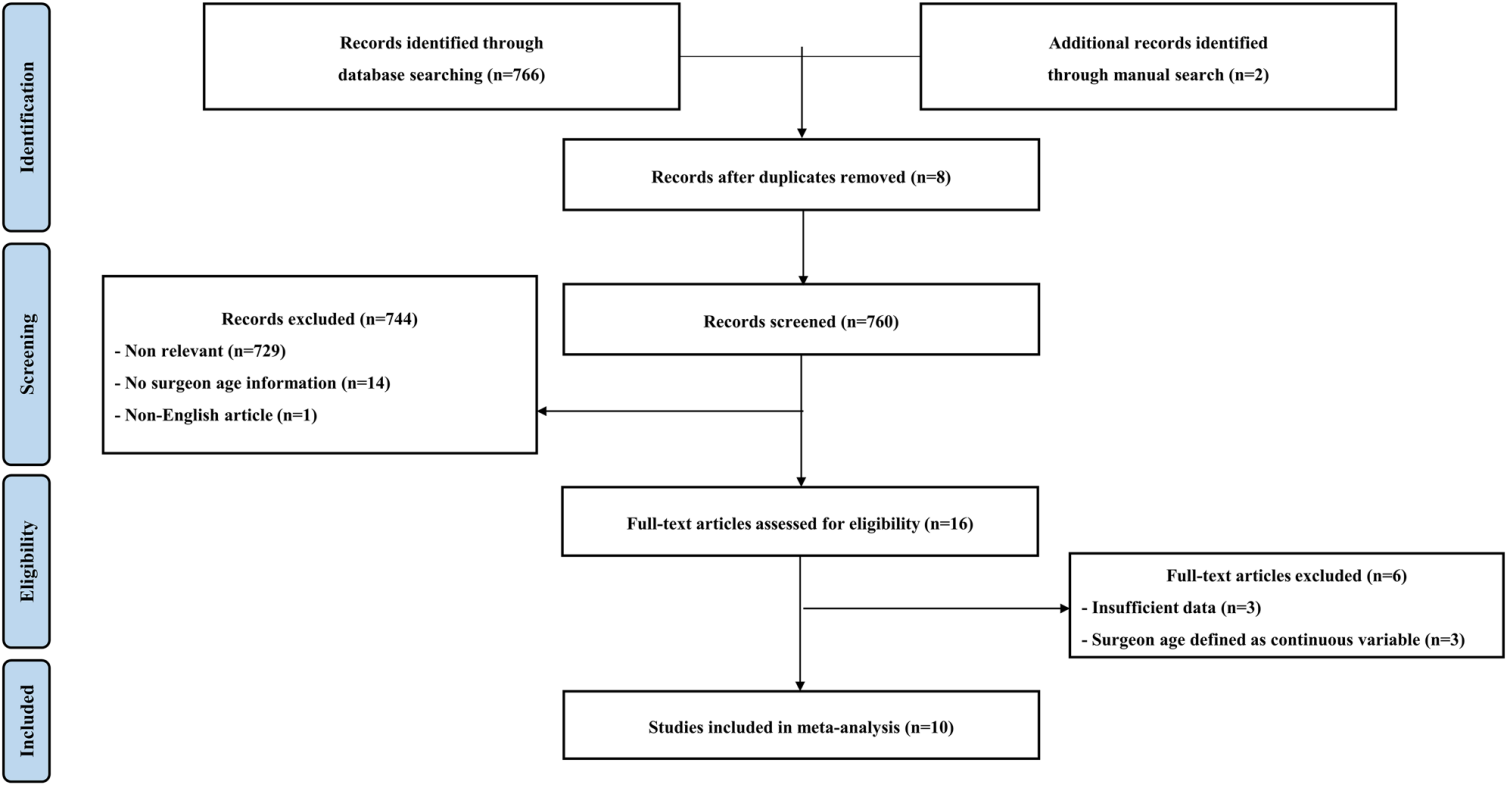
The PRISMA flow diagram.

A total of 760 records were screened based on their title and abstract. A full-text review of 16 papers was conducted, and a total of 10 cohort studies were finally chosen (Fig. 1)^7,14–22^. The characteristics of the included studies are listed in Table 1.

**Table 1:** Characteristics of the studies included for the analyses.

	Study, year	Outcomes	Surgeon’s age	Types of surgery	Patients studied (n)	Nationality	Study period	Major morbidity	Adjustment
1	Matar et al.^14^	Unadjusted OR (morbidity) Adjusted OR (morbidity)	< 45 years (young) 45–55 years (middle) > 55 years (old)	Total hip arthroplasty	Total—122,043 Young—47,726 Middle—35,842 Old—38,475	Canada	2002–2018	Composite complication	Clustering by surgeon
2	Lin et al.^15^	Unadjusted OR (morbidity)	28–41 years (young) 41–50 years (middle) 50–65 years (old)	Adenoidectomy	Total—5435 Young—3439 Middle—1522 Old—474	Taiwan	2002–2011	Reoperation	
3	Tsugawa et al.^16^	Unadjusted OR (mortality) Adjusted OR (mortality)	< 40 years (young) 40–49 years (middle) 50–59 years (old) ≥ 60 years (old)	Twenty major surgical procedures (16 most common non-cardiovascular surgeries in Medicare population and 4 common cardiovascular surgeries)	Total—892,187 Young—149,349 Middle—292,103 Old—450,735	USA	2011–2014		Patients’ and surgeons’ characteristics and hospital fixed effects
4	Anderson et al.^7^	Unadjusted OR (morbidity) Unadjusted OR (mortality) Adjusted OR (morbidity) Adjusted OR (mortality)	< 40 years (young) 40–50 years, (middle) 50–60 years (old) > 60 years (old)	Congenital heart surgery	Total—62,851 Young—6198 Middle—29,391 Old—27,262	USA	2010–2014	Major morbidity	Not specified
5	Markar et al.^17^	Unadjusted OR (morbidity) Unadjusted HR (mortality) Adjusted HR (mortality)	≤ 51 years (young) 52–55 year (middle) ≥ 56 years (old)	Esophagectomy	Total—1761 Young—946 Middle—291 Old—524	Sweden	1987–2010	Reoperation	Age, sex, comorbidity, tumor stage, tumor histology, neoadjuvant therapy, surgeon volume of esophagectomies, and calendar period of surgery
6	Stevens et al.^18^	Unadjusted OR (morbidity) Adjusted OR (morbidity)	45 years (young) 45–55 years (middle) > 55 years (old)	Primary laparoscopic Roux-en-Y Gastric Bypass, sleeve gastrectomy	Total—60,430 Young—14,322 Middle—31,936 Old—14,172	USA	2006–2016	Overall complication	Patient characteristics and comorbidities, and surgeon volume, years of experience, a and fellowship
7	Wu et al.^19^	Unadjusted OR (morbidity) Adjusted OR (morbidity)	< 40 years (young) 40–49 year (middle) ≥ 50 years (old)	Hysteropexy and hysterectomy	Total—36,609 Young—9256 Middle—17,011 Old—10,342	Taiwan	1997–2010	Repeat surgery	Not specified
8	Ho et al.^20^	Unadjusted OR (morbidity) Adjusted OR (morbidity)	≤ 40 years (young) 41–50 year (middle) ≥ 51 years (old)	Scleral bucking, pars plana vitrectomy, or both	Total—7427 Young—2994 Middle—3668 Old—765	Taiwan	2002–2004	180-day readmission	Surgeon volume, hospital volume, and hospital level
9	Waljee et al.^21^	Adjusted OR (mortality)	≤ 40 years (young) 41–50 years (middle) 51–60 years > 61 years (old)	Eight procedures (coronary artery bypass grafting, elective abdominal aneurysm repair, aortic valve replacement, carotid endarterectomy, pancreatectomy, esophagectomy, lung resection, and cystectomy)	Total—461,000	USA	1998–1999		Patient and provider characteristics
10	O’Neill et al.^22^	Unadjusted OR (morbidity) Unadjusted OR (mortality)	30–39 years (young) 40–49 years (young) 50–59 years (middle) 60–64 years (old) 65 or higher (old)	Carotid endarterectomy	Total—11,424 Young—7438 Middle—2931 Old—1055	USA	1994–1995	Number of bad outcomes	

### Synthesis of results

#### Mortality

**Figure 2:**
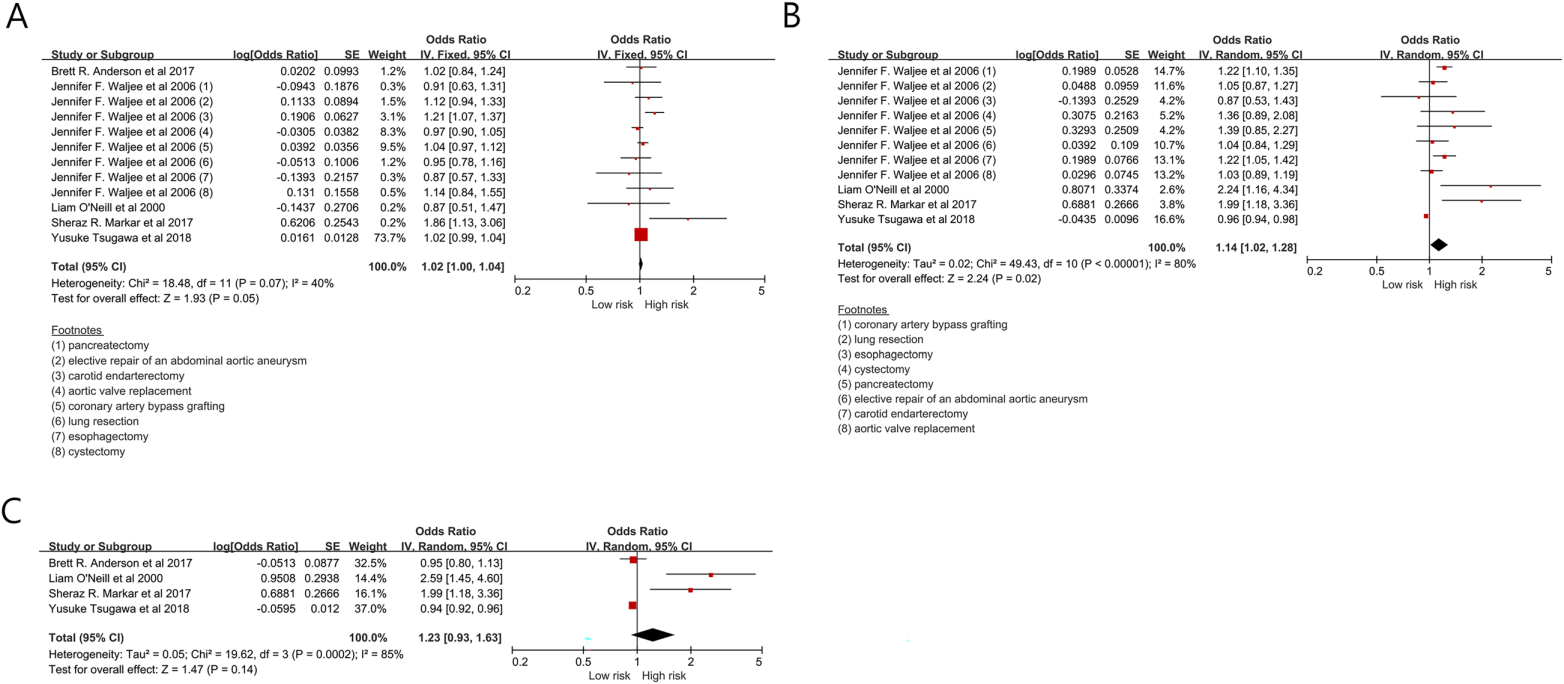
The mortality in the patients undergoing surgery according to surgeon’s age.

The mortality in patients undergoing surgery by young surgeons was 1.02 (1.00–1.04, *p* = 0.05) (I^2^ = 40%) compared to those by middle-aged surgeon (Fig. 2A). The mortality in patients undergoing surgery by old-aged surgeons was 1.14 (1.02–1.28, *p* = 0.02) (I^2^ = 80%) compared to those by middle-aged surgeon (Fig. 2B). The mortality in patients undergoing surgery by old-aged surgeons was 1.23 (0.93–1.63, *p* = 0.14) (I^2^ = 85%) compared to those by young surgeon (Fig. 2C).

#### Major morbidity

**Figure 3:**
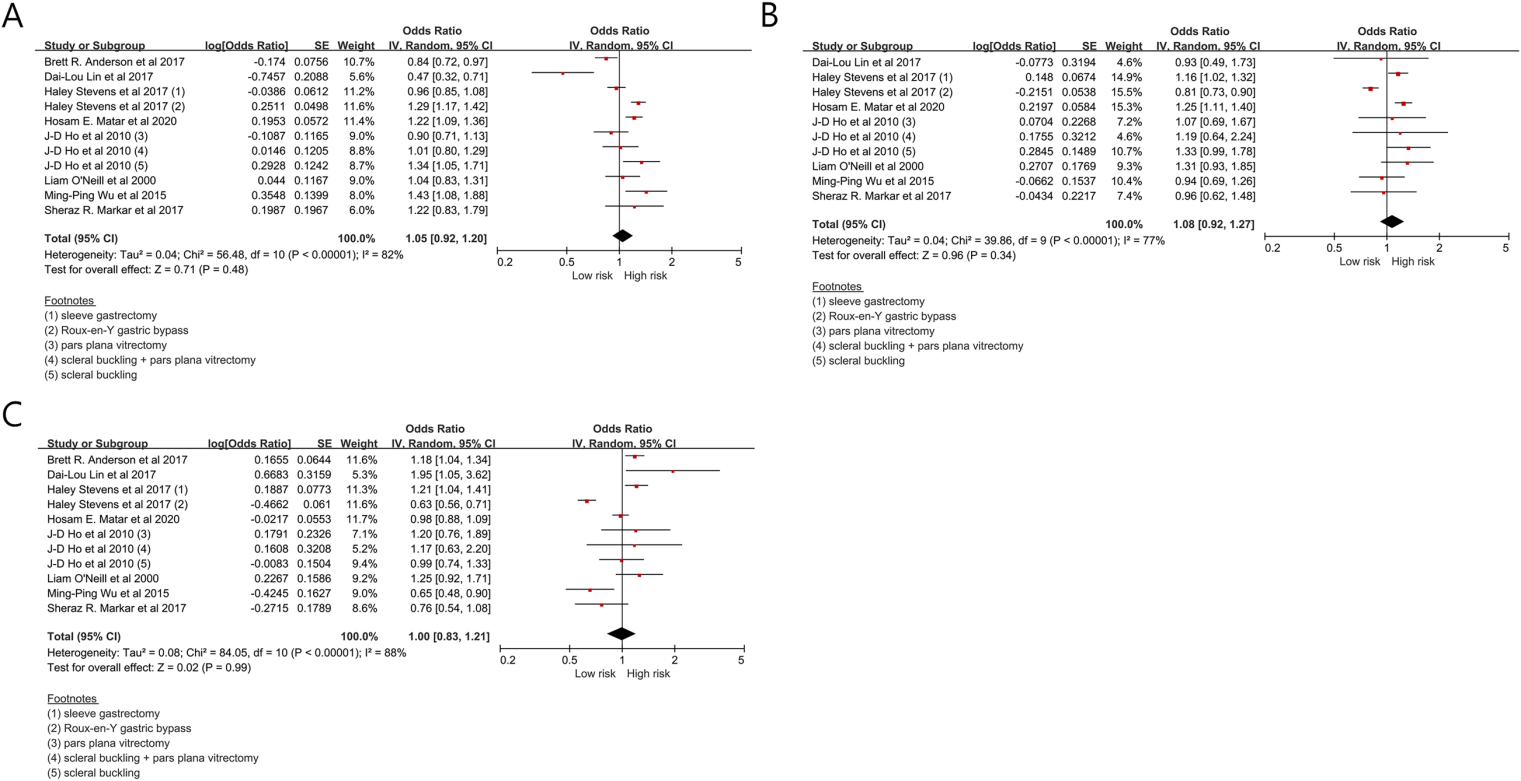
The major morbidity in the patients undergoing surgery according to surgeon’s age.

The major morbidity in patients undergoing surgery by young surgeons was 1.05 (0.92–1.20, *p* = 0.48) (I^2^ = 82%) compared to those by middle-aged surgeon (Fig. 3A). The major morbidity in patients undergoing surgery by old-aged surgeons was 1.08 (0.92–1.27, *p* = 0.34) (I^2^ = 77%) compared to those by middle-aged surgeons (Fig. 3B). The major morbidity in patients undergoing surgery by old-aged surgeons was 1.00 (0.83–1.21, *p* = 0.99) (I^2^ = 88%) compared to those by young surgeons (Fig. 3C).

We performed subgroup analysis of major morbidity according to major and minor surgeries. In subgroup analysis, no significant difference in major complications was found according to the age difference of surgeons. A detailed analysis of the results was provided in Table 2.

**Table 2:** Subgroup analysis of major morbidity stratified by major and minor surgery.

Major morbidity	Number of results	Heterogeneity (%)	Odds ratio (95% confidence interval)
**Major surgery**			
Young (ref. middle)	7	81	1.10 (0.96–1.26)
Old (ref. middle)	6	81	0.96 (0.81–1.13)
Old (ref. young)	7	92	0.92 (0.74–1.16)
**Minor surgery**			
Young (ref. middle)	4	84	0.90 (0.65–1.26)
Old (ref. middle)	4	0	1.20 (0.97–1.48)
Old (ref. young)	4	21	1.15 (0.92–1.42)

### Risk of bias within the studies

According to Newcastle–Ottawa criteria, six out of ten cohort studies were rated as 'good', while four were rated as ‘poor’. Detailed assessments of the risk of bias have been represented in Table 3.

**Table 3:** Quality assessment of included studies.

Study	Selection				Comparability based on design and analysis	Outcome			Total	Assessment
	Representativeness of the sample	Selection of the non-intervention cohort	Ascertainment of exposure	Demonstration that outcome of interest was not present at the start of the study		Assessment of outcome	Was follow-up long enough for outcomes to occur	Adequacy of follow-up of cohorts		
Matar et al.^14^	1	1	1			1	1	1	6	Poor
Lin et al.^15^	1	1	1			1	1	1	6	Poor
Tsugawa et al.^16^	1	1	1	1	2	1	1		8	Good
Anderson et al.^7^	1	1	1	1	2	1	1		8	Good
Markar et al.^17^	1	1	1		2	1	1	1	8	Good
Stevens et al.^18^	1	1	1	1	2	1		1	8	Good
Wu et al.^19^	1	1	1			1	1		5	Poor
Ho et al.^20^	1	1	1	1	2	1	1	1	9	Good
Waljee et al.^21^	1	1	1	1	2	1		1	8	Good
O’Neill et al.^22^		1	1	1		1	1		5	Poor

Good quality: 3 or 4 stars in selection domain AND 1 or 2 stars in compatibility domain AND 2 or 3 stars in outcome/exposure domain, Fair quality: 2 stars in selection domain AND 1 or 2 stars in comparability domain AND 2 or 3 stars in outcome/exposure domain, Poor quality: 0 or 1 star in selection domain OR 0 stars in comparability domain OR 0 or 1 stars in outcome/exposure domain.

## Discussion

This meta-analysis combines the data from 10 retrospective cohort studies examining the association between surgeons’ age and mortality and morbidity after various surgeries. With a total of 1,666,108 patients and 29 kinds of surgery, this is the largest body of information and first meta-analysis, so far available, for evaluating the effect of the surgeons’ age on the postoperative outcomes.

This meta-analysis established that the surgeries performed by old-aged surgeons incurred higher mortality than those by middle-aged surgeons. Although there were instances of increased mortality in the case of the surgeries performed by young surgeons than those by middle-aged surgeons, they were not statistically different. The studies that analysed the postoperative mortality between middle-aged and older surgeons after several surgical procedures, like coronary artery bypass grafting (CABG), carotid endarterectomy (CEA), and esophagectomy, showed higher mortality when they were performed by old-aged surgeons. The major morbidity did not differ according to the age of the surgeons. As the nature of the surgical procedures evident in the studies for major morbidity were mixed, these surgeries were subdivided into large-organ surgeries called major surgeries and small-organ surgeries called minor surgeries. Our analysis showed that the morbidities did not differ according to the surgeons’ age in the case of both the major and minor surgeries.

The surgical workforce, like the rest of the population, is ageing^23^. In the US, nearly one-third of the active surgeons are older than 55 years^24^. Similarly, in Australia, the average age of a surgeon is 52 years, and 19% of the active surgeons are at least 65 years or older^25^. Like everyone, surgeons also undergo age-related deterioration of the neurocognitive, sensory, and motor functions^26^. There are predictable age-related degenerations across several areas of cognitive function, like diminished processing speed, clinical reasoning, and adaptive thinking^26,27^. This delays the ability of decision-making, which is critical for surgeons^28^. Physical activity slows down with age and movements become less integrated with cognitive thinking; therefore, older surgeons respond more slowly when the task of decision-making is involved^29,30^. The effect of ageing on hand dexterity is of obvious importance to surgeons. Hand function and manual dexterity diminish with ageing, such that the ability to control the force with each finger undergoes deterioration^30^. Previous studies have demonstrated that the strength, visuospatial ability, cognitive skills, and abilities to sustain attention decrease with age^28,31,32^. This may be critical to some procedures requiring a high degree of precision and small anastomoses, such as CABG, CEA, and pancreatectomy.

As surgical specialities advance rapidly, the ageing surgeons may struggle to keep up and reluctantly incorporate new techniques^33^. Moreover, the remoteness between ending formal education and current practice is considerable for the ageing surgeon. In the treatment of melanoma, the older surgeons prescribed more chemical tests that are no longer believed to be helpful in the treatment of melanoma^34^. Similarly, the older surgeons were less likely to perform immediate reconstruction as they believed that immediate breast reconstruction had disadvantages^35^. Choudhury et al.^36^ found a negative relationship between the physician’s age and adherence to the standard of therapeutic care. Considering this finding, it is not surprising that the older surgeons have inferior performance in the recertification examinations^37^. In light of the fact that clinical guidelines and standards of practice are critical for patient safety and change periodically based on evidence, not following them may prove problematic^38^.

It has been an arguable issue for a long time whether these changes in the physiology and clinical patterns with age is correlated with a patient’s outcome and there should be a recertification programme for old surgeons. A growing body of literature represents that more experienced surgeons have worse clinical outcomes paradoxically and raised concerns on the need for mandatory retirement age or recertification programmes for old surgeons^36,39,40^. Contrarily, a recent study by Tsugawa et al.^16^ showed that the patients’ mortality was lower for older surgeons than for younger surgeons, which was included in our meta-analysis. They tried to minimise the bias on analysis by including only emergency surgeries, thus avoiding the patients’ selection on the surgeons with age and surgeons’ selection of patients based on the severity of illness. They calculated the OR of the postoperative mortality of patients who underwent surgeries by old-aged surgeons as a reference to surgeons aged under 40. However, we considered the mid-career surgeons as the surgeons of age over a minimum of 40 years and more suitable as a reference age to analyse the old-aged surgeons’ mortality OR. With this strategy, our meta-analysis also showed that the mortality of old surgeons was higher than that of middle-aged surgeons, suggesting that the postoperative mortality curve is convex according to the surgeon’s age.

Senior surgeons are unarguably an invaluable asset to surgical societies. With their lifetime experience of surgeries and clinical cares, they have provided mentorship to future generations of surgeons, impacted the scientific literature, and led to advances in surgical skill. As age-related physiological changes and clinical patterns are highly variable between the individuals, a multidisciplinary approach to evaluate the performance of old-aged surgeons should be performed by professional organisations, not by chronological age, which is usually applied to pilots in the airline industry.

This study has some limitations. First, the studies included in this meta-analysis were retrospective. Second, there were heterogeneities in the types of surgeries and definitions of surgeon’s age. Third, complicated and difficult surgeries were more likely to be performed by older surgeons than by younger ones. Fourth, the surgeries on the analysis of mortality and morbidity were not matched. For this, we subdivided the surgeries into major and minor surgeries for analysing the morbidity; however, the number of major surgeries were few. For this reason, we could not interpret the connection results from the mortality and major morbidity with the surgeons’ age. Lastly, the included studies were mostly drawn from administrative datasets, such as the Medicare and National Health Insurance Research Dataset. It is difficult to find causal mechanisms of postoperative mortality in these datasets. Future research using individual-level data including the length of procedure, length of hospital stays, and postoperative complication rates are needed to address the assess surgeon’s outcome accurately.

## Conclusion

In conclusion, this meta-analysis indicates that the surgeons’ ageing increased the risk of postoperative mortality, but not of major morbidity. Although the underlying mechanism was not determined as they included only cohort studies, our results provide evidence necessitating the introduction of a multidisciplinary approach to evaluate the performance of the senior surgeons.

## Supplementary Information

Supplementary Information.

## Data Availability

The datasets used and/or analysed during the current study available from the corresponding author on reasonable request.
